# Development of a patient-reported outcome measure for patients who have recovered from a subarachnoid hemorrhage: the “questionnaire for the screening of symptoms in aneurysmal subarachnoid hemorrhage” (SOS-SAH)

**DOI:** 10.1186/s12883-021-02184-x

**Published:** 2021-04-16

**Authors:** Edith Nobels-Janssen, Inger L. Abma, Wim I. M. Verhagen, Ronald H. M. A. Bartels, Philip J. van der Wees, Jeroen D. Boogaarts

**Affiliations:** 1 Department of Neurology, Canisius Wilhelmina Hospital, Nijmegen, the Netherlands; 2 Department of Neurosurgery, Radboud University Medical Center, HB 6500 Nijmegen, the Netherlands; 3 IQ Healthcare and Rehabilitation, Radboud University Medical Center, Radboud Institute for Health Sciences, Nijmegen, the Netherlands

## Abstract

**Background:**

Patients who have been successfully treated for an aneurysmal subarachnoid hemorrhage (aSAH) often retain multiple health complaints, including mood disorders, cognitive complaints, fatigue, and problems with social participation. These problems are not always fully addressed during hospital visits or in current outcome measures, such as the modified Rankin score and the Glasgow Outcome Scale. Here, we present the development of the “Questionnaire for the Screening of Symptoms in aneurysmal Subarachnoid Hemorrhage” (SOS-SAH), which screens for the self-reported symptoms of patients with mild disabilities.

**Methods:**

During the development of the SOS-SAH we adhered to the PROM-cycle framework for the selection and implementation of patient-reported outcome measures (PROMs). The SOS-SAH was developed in an iterative process informed by a literature study. Patients and healthcare professionals were involved in the development process through participating in a working group, interviews, and a cognitive validation study.

**Results and conclusions:**

Relevant patient-reported outcomes (PROs) were identified for patients with aSAH. The SOS-SAH was developed primarily using domains and items from existing PROMs and, if necessary, by developing new items. The SOS-SAH consists of 40 items and covers 14 domains: cognitive abilities, hypersensitivity to stimuli, anxiety, depression, fatigue, social roles, personality change, language, vision, taste, smell, hearing, headache, and sexual function. It also includes a proxy measurement for use by family members to assess cognitive functioning and personality change.

## Key points

- Several symptoms often remain undetected after an aneurysmal subarachnoid hemorrhage (aSAH). A disease-specific PROM for aSAH might reveal more non-spontaneously mentioned symptoms.

- The SOS-SAH was developed to cover relevant PROs for patients successfully treated after an aSAH.

- The SOS-SAH consists of 40 items covering 14 domains, and an additional section for a proxy measurement.

## Background

An aneurysmal subarachnoid hemorrhage (aSAH) is the result of a rupture of an intracranial aneurysm resulting in an accumulation of blood in the subarachnoid space. The incidence rate is 6.1 per 100,000 person-years [1]. Advances in the management of aSAH have led to a mortality rate reduction of 17% over the last 30 years [2–4], meaning the daily functioning and quality of life of patients who survived an aSAH is therefore becoming more and more important.

The typical patient suffering an aSAH is relatively young (mean age 50–55 years), meaning this condition has a high impact on society through a loss of productive life years [5, 6]. The estimated costs of an aSAH over a patient’s lifetime are twice as high as the costs of an ischaemic stroke, mainly caused by the younger age of patients with aSAH and the higher mortality rate [7]. Approximately half of patients with a successfully treated aSAH experience problems with memory, mood, or neuropsychological function, and a third have problems in societal participation [8–10]. This highlights the need to measure these problems from a patient’s point of view using patient-reported outcomes (PROs). These PROs can be assessed using patient-reported outcome measures (PROMs), which are questionnaires consisting of one or multiple items. Healthcare professionals can use PROMs in clinical practice to focus on a patient’s individual health status and as an aid in the follow-up care of patients. PROMs can also be used across multiple patients for a quality assessment of treatment and for public reporting to promote external transparency [11, 12].

A limited number of validation studies have evaluated PROMs in patients with aSAH [13]. Due to a low level of evidence for the quality of these PROMs, no evaluation of content validity or unclear phrasing of items, none of the cited PROMs were deemed suitable for use in clinical practice. We therefore describe the development process of a new PROM, the “Questionnaire for the Screening of Symptoms in aneurysmal Subarachnoid Hemorrhage” (SOS-SAH), which was conducted in collaboration with patients, healthcare professionals, and specialists in PROM development. Our aim was to develop a PROM for use in individual patients with aSAH to guide their follow-up care, especially for those patients with mild disabilities that have an apparent good outcome but may suffer from symptoms that they often do not actively mention.

## Methods

### Design and setting

**Fig. 1:**
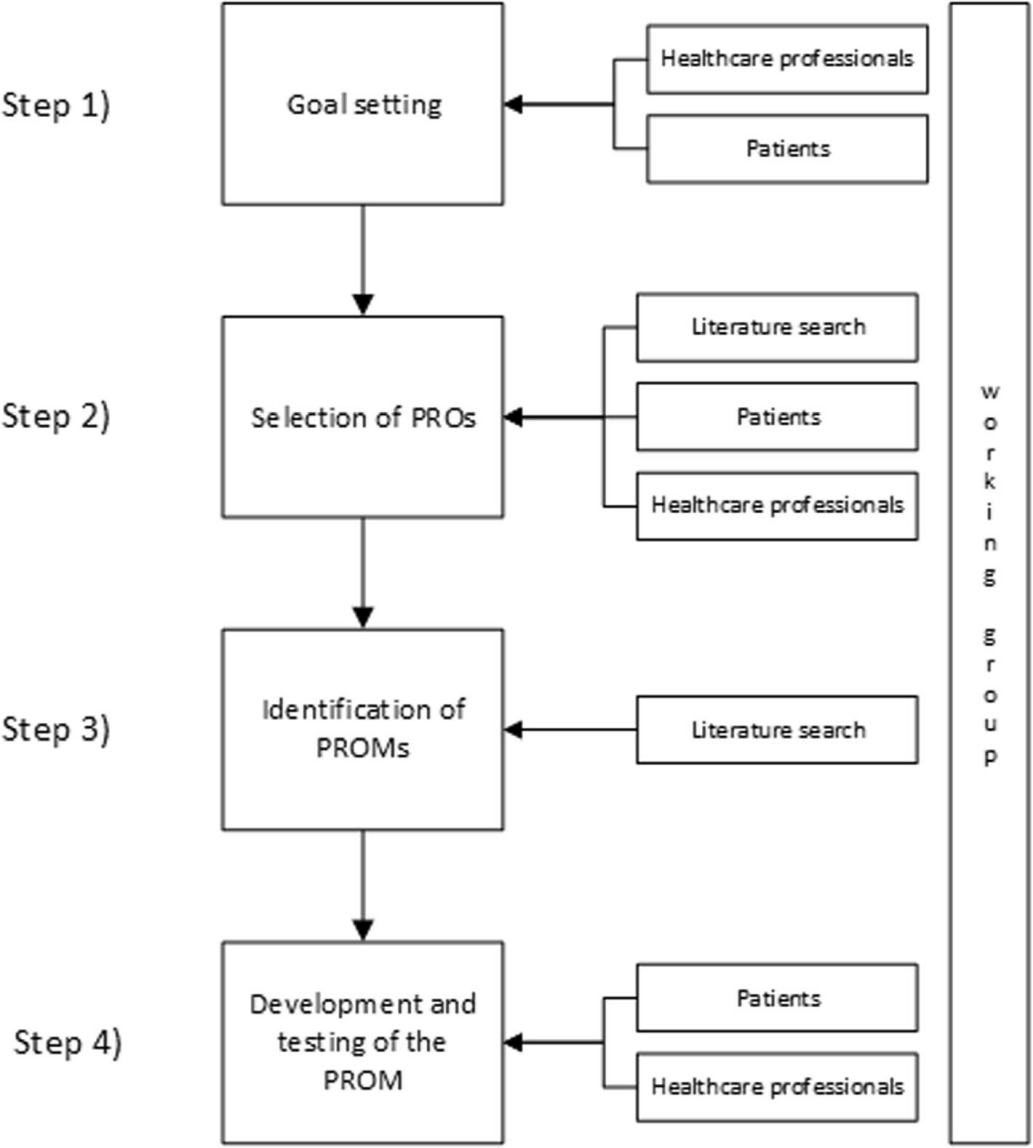
Flowchart of the steps of the PROM-cycle [14] and involved parties and processes in the development of the SOS-SAH. PROs: patient-reported outcomes; PROMs: patient-reported outcome measures

This study was conducted in the Netherlands from May 2018 to April 2020. We adhered to the “PROM-cycle”, a framework with eight steps necessary for the selection and implementation of PROMs in healthcare [14]. The first four steps that focus on the development of a PROM are described: (1) goal setting for the PROM, (2) selection of important PROs, (3) identification of PROMs that cover the selected PROs, and (4) the development and testing of a PROM. Throughout the development process, collaboration with patients and healthcare professionals was assured through iterative interactions. The involvement of the different parties and processes in each step of the PROM-cycle is presented schematically in Fig. 1.

#### Scope

The goal of the PROM is to screen individual patients with mild disabilities who have been successfully treated for aSAH, since they are prone to undetected symptoms during their follow-up care. “Mild disabilities” was defined as an outcome on the modified Rankin Scale (mRS) of 0, 1, or 2. The mRS is a global disability scale that has an ordinal scale ranging from no residual symptoms (score of 0) to severely disabled (score of 5) [15]. The intention was to construct the PROM using (the domains of) existing PROMs and, if necessary, newly developed items.

### Procedures

#### Working group

The working group included a lead researcher (EN), two researchers experienced with developing PROMs (IA and PW), a patient who has experienced aSAH (AS, a member of the patient organization of patients with an intracranial aneurysm hosted at Radboud university medical center), and a neurosurgeon (JB). The working group made the necessary decisions during the development process of the SOS-SAH.

#### Interviews with healthcare professionals

A focus group interview was scheduled with healthcare professionals involved in the acute in-hospital care, as well as follow-up and rehabilitation treatment, of patients with an aSAH, which informed step 1 of the PROM-cycle (goal setting), step 2 of the PROM-cycle (selection of PROs), and an exploration of some aspects of step 4 of the PROM-cycle (development and testing of the PROM). Neurologists, neurosurgeons, rehabilitation physicians, and specialized nurses from different regions in the Netherlands were contacted to participate in a focus group interview. Five healthcare professionals (Table 1) participated in the focus group interview, led by an experienced moderator. As a second step, two individual interviews were conducted with a neurologist and with a rehabilitation specialist who could not attend the focus group interview. The following topics were discussed: the goal setting for the PROM, the relevant PROs for patients with an aSAH, the ideal timing for completion of the PROM, and the best strategy for the visual representation of the results of the PROM. Afterwards there were no unresolved issues, and a shared general view of healthcare professionals involved with the care for patients with aSAH in the hospital setting was achieved. The semi-structured interviews were recorded and transcribed verbatim. A thematic analysis was performed on the data using ATLAS.ti version 8.4.20. The interviews were coded based on a topic list, and new topics that emerged during the analysis were added to this list. Coding was performed independently by two researchers (EN and IA), and differences in the codes were discussed by these two researchers until a consensus was reached. A summary of the interviews was returned to the participants for comments or corrections.

**Table 1:** The characteristics of the interviewees

	Health care professionals n (%)	Patients round one n (%) or median (range)	Patients round two n (%) or median range
Total number of participants	7 (100%)	11 (100%)	7 (100%)
Function			
Medical doctor	6 (85.7%)	n.a.	n.a.
Nurse practitioner	1 (14.3%)	n.a.	n.a.
Medical specialty			
Neurology	2 (28.6%)	n.a.	n.a.
Neurosurgery	4 (57.1%)	n.a.	n.a.
Rehabilitation medicine	1 (14.3%)	n.a.	n.a.
Age (mean, range)	49 (30–61)	60 (43–86)	53 (25–74)
Gender			
Male	4 (57.1%)	5 (45.5%)	4 (57.1%)
Female	3 (42.9%)	6 (54.5%)	3 (42.9%)
modified Rankin Scale score	n.a.	2 (0–3)	n.a.
Years after aSAH (mean, range)	n.a.	3 (1,5-4,0)	3 (1,5-7,0)

#### Literature search

The literature was screened to identify relevant PROs for patients with aSAH that could be related to symptoms, activities, and participation, according to the International Classification of Functioning, Disability, and Health (ICF) model [16]. This is a framework for describing and organizing information on functioning and disability and conceptualizes a person’s level of functioning as an interaction between health conditions, environmental factors, and personal factors.

The validity of the available PROMs for patients after aSAH was evaluated in a recent systematic review [13]. We used this review to select the PROs and PROMs for this study. Additionally, we searched PubMed for qualitative studies about the outcomes in patients after aSAH (using a qualitative research filter (MeSH term) and keywords “subarachnoid hemorrhage” and “outcome”), and performed a search guided by articles and their references previously included in the national guideline.

For all PROs identified in the literature, we searched for PROMs that captured the outcome and assessed its content validity (including face validity) and the length of the questionnaire. Information on structural validity was used to assess the dimensionality of multi-domain PROMs. Only unidimensional domains were considered potentially suitable to be part of the new PROM.

#### Interviews with patients: round one

Eleven patients from three hospitals in the Netherlands were interviewed (Table 1) by trained interviewers to cover step 2 of the PROM-cycle (selection of PROs), and step 3 of the PROM-cycle (identification and selection of PROMs). These patients were successfully treated for an aSAH less than five years ago, and were selected by their treating physician. They randomly selected patients of different ages, genders, and mRS outcome scores. The topics covered in the interviews were: which PROs are relevant for patients with aSAH, the potential value of using a PROM in their own healthcare following aSAH treatment, the acceptable number of questions for the PROM to keep the questionnaire complete but concise, and the preference of completing a questionnaire on paper or online. The goal of the interview was not to perform an explorative study about all possible PROs experienced by patients successfully treated for aSAH, but more specifically to confirm the relevance of previously detected PROs in the literature and by healthcare professionals, and to discuss any other relevant PROs. The interviewees were asked about the content and comprehensiveness of several existing PROMs, i.e., the domains of the Stroke Specific Quality of Life scale (SS-QoL) [17], the Cognitive and Emotional Consequences of Stroke (CLCE-24) [18], the Hospital Anxiety and Depression Scale (HADS) [19, 20], the Quality of Life after Brain Injury scale (QOLIBRI) [21], the Patient-Reported Outcomes Measurement Information System questionnaire (PROMIS) [22], and the Stroke Impact Scale (SIS) [23]. These PROMs were selected because they have been (partly) validated for use in patients with aSAH (as found in the systematic review [13]) or commonly used in research studying the outcomes of aSAH.

The interviews were recorded, and a verbatim transcript was made. A thematic analysis with a directed approach was performed based on a topic list guided by the interview script. The analysis was performed in ATLAS.ti. Any new themes that emerged during the analysis were added to this topic list.

#### Interviews with patients: round two

After developing the preliminary PROM, a cognitive validation study was conducted to evaluate whether the questions of the SOS-SAH were interpreted in the way they were intended (step 4 of the PROM-cycle: testing of the PROM). This enabled the evaluation of whether the selected (domains of) existing PROMs were suitable for use in patients with aSAH, and to assess the comprehensibility of any newly formulated items. Patients with a successfully treated aSAH who attended the outpatient clinic were asked to participate. During the interview, the patients completed the SOS-SAH and shared their thoughts about the purpose and phrasing of the questions and about the comprehensiveness of the SOS-SAH [24]. An interview guide was used for all the interviews, which were transcribed verbatim. The analysis was performed based on a topic list and any new issues that emerged during the analysis were added to this topic list. Based on the results, the working group made the final decision about the removal or adaptation of unclear items, leading to the final version of the SOS-SAH.

## Results

### Goal setting

The main goal of the SOS-SAH is to screen for undetected symptoms in individual patients with mild disabilities following their successful treatment for aSAH. Healthcare professionals stated that complaints and mild disabilities common in patients with aSAH are not always recognized during regular hospital visits, in contrast to the complaints of patients with higher mRS values. Patients with mild disabilities may not actively mention these symptoms during the follow-up consultation, and due to time restraints healthcare professionals also may not inquire about all of them. This prohibits potential treatment or therapy. Healthcare professionals mentioned the following symptoms that might not be recognized: fatigue, problems with cognitive functioning, anxiety, depression, barriers in social participation, and headaches.

According to patients, a PROM might help them to identify and bring up the topics they want to discuss during a consultation, as well as providing insight into which of their complaints might be caused by the aSAH. However, none of the patients thought that the use of a questionnaire in their own care would have had a major impact on the healthcare they received. Despite that, half of the interviewed patients have the feeling they lacked something in their follow-up care, such as support or more timely and regular follow-up appointments.

All interviewed patients and healthcare professionals were in favor of an additional proxy measurement, which is a measurement completed by a person close to the patient. This would facilitate the indirect measurement of certain domains of functioning, which is especially important in patients with a decreased insight into their own performance due to brain damage, as can be the case in patients with aSAH, which was also found to be relevant in patients with an apparently good outcome.

### Selection of PROs

In the literature, 17 PROs were identified for patients with aSAH, listed in Table 2 [3, 9, 25–27]. The working group decided that if the PROs showed a major overlap, e.g., fatigue and sleep disorders, or post-traumatic stress disorder and anxiety, only one PRO was to be included in the PROM. Healthcare professionals and patients considered cognitive functioning, depression, anxiety, fatigue, social roles, and personality to be the most important PROs for the SOS-SAH.

**Table 2:** The selection process of relevant PROs for patients with an aSAH

PROs	Identified from literature (L) or added after interviews with healthcare professionals or patients (P)	Final PROs used in the SOS-SAH
Fatigue	L	Yes
Pain / headache	L	Yes (headache only)
Mobility	L	No
Self-care	L	No
Vision	L	Yes
Language	L	Yes
Upper extremity functioning	L	No
Sleep disorders	L	No
Problems with sexuality	L	Yes
Cognitive functioning	L	Yes
Personality change	L	Yes
Depression	L	Yes
Anxiety	L	Yes
Posttraumatic stress disorder	L	No
Work and productivity	L	No
Family roles	L	No
Social roles	L	Yes
Smell	P	Yes
Hearing	P	Yes
Taste	P	Yes
Hypersensitiviy to stimuli	P	Yes
Degree of recovery	P	No

L: literature; P: healthcare professionals or patients; PROs: patient-reported outcomes; SOS-SAH: Questionnaire for Screening of Symptoms in aneurysmal Subarachnoid Hemorrhage

The healthcare professionals recommended the addition of the following to the SOS-SAH: smell, taste, hearing, headache (but not pain in general), and hypersensitivity to especially visual and acoustic stimuli (Table 2). Hypersensitivity to stimuli was also frequently mentioned by the patients. Sexual dysfunction is not frequently mentioned as an important outcome after aSAH [28], but healthcare professionals recommended the active exploration of this outcome, which patients may otherwise be reluctant to present. The working group excluded PROs that represent complaints on the functional level, such as mobility and upper extremity function, since they are not the main focus of this study (i.e., they are usually detected in regular assessments and evaluation).

### Identification of PROMs

Only a few validation studies have assessed PROMs specifically for patients with aSAH, and generally these are of low quality [13]. The most suitable PROM currently available for patients with aSAH is the SS-QoL [13]; however, this PROM lacks an assessment of content validity, and the assessment of structural validity resulted in two factors and not the proposed twelve domains that the SS-QoL aims to measure [13]. This makes the validity of the separate domain scores questionable. We identified one disease-specific PROM for patients with aSAH: the Subarachnoid Haemorrhage Outcome tool (SAHOT) [29]. The SAHOT consists of 56 items that patients rate on a three-point scale according to the change they experienced. Since no direction of change (better or worse) is incorporated in the answering options, the working group considered the face validity of this tool to be insufficient. The development and assessment of its measurement properties were of low quality [13]. The interpretation of the SAHOT was developed using a Rasch-based interval analysis (which is based on item response theory and requires time for a digital analysis of the answers using Rasch models), which makes it unfeasible for use in daily practice. The SS-QoL and the SAHOT were therefore considered unsuitable for inclusion in the current PROM.

Since the validated PROMs for patients with aSAH were not suitable, we searched the literature for more generic PROMs to capture the selected PROs. The selected PROMs were the CLCE-24 [18], the HADS [19, 20], QOLIBRI questionnaire [21], the PROMIS questionnaires [22], and the SIS [23]. We selected these PROMs because they are (at least partially) validated for use in patients with aSAH, have previously been used in research into the outcomes of aSAH, or because they are broadly accepted PROMs.

### Development and testing of the PROM

#### Number of questions

Both healthcare professionals and patients found 30–40 items to be the acceptable maximum number in the PROM; despite this, some patients reported that in practice they would complete the PROM in multiple attempts due to a lack of energy and/or concentration. The PROM consists of multiple questions addressing the most important PROs; cognitive functioning, anxiety, depression, fatigue, and social roles. In order to keep the SOS-SAH relatively brief, only one or two questions were used to address the other PROs.

#### Patients’ preferences

The interviewed patients in general preferred PROMs that adhere to their actual experiences and feelings, and preferred positively formulated items; for example, ‘I feel cheerful’ (HADS) was preferred over ‘I felt worthless’ (PROMIS short form for depression)). Healthcare professionals advised the inclusion of a question about the degree of recovery after the aSAH; however, during pilot testing, several patients interpreted this question as asking whether the aneurysm itself was healed, which was not the aim of the question. Additionally, only one patient could provide an estimated degree of recovery, because the concept of general recovery did not capture the different persisting complaints or because complaints fluctuated daily. The working group therefore decided not to add this item to the PROM.

#### Preliminary SOS-SAH

After consensus was reached in the working group, the SOS-SAH was composed of the HADS, the PROMIS short form for fatigue, the PROMIS short form for social roles, a customized PROMIS short form for cognitive abilities, and 10 questions to cover the other nine PROs (Table 3). The HADS is a questionnaire that measures anxiety and depression, with seven questions per domain [19]. PROMIS HealthMeasures develops item banks for frequently assessed PROs that are validated for use in the general population and for persons with chronic diseases. Ideally, these databases are used as computer adaptive tests (CATs); however, since CATs are not yet feasible for widespread use in Dutch clinical practice and because patients with aSAH preferred to complete the PROM in paper form, we opted to use the short forms that were created based on the item banks [22].

**Table 3:** Used PROMs to cover the identified PROs

PROs used in the PROM	PROMs
Cognitive abilities	PROMIS 8-item custom short form
Depression	HADS
Anxiety	HADS
Fatigue	PROMIS 4-item short form
Social roles	PROMIS 4-item short form
Hypersensitivity to stimuli	Adapted question from the QOLIBRI
Personality change	Adapted question from the QOLIBRI
Language	Adapted questions from the QOLIBRI
Vision	Adapted question from the QOLIBRI
Taste	Adapted question from the QOLIBRI
Smell	Adapted question from the QOLIBRI
Hearing	Adapted question from the QOLIBRI
Headache	Adapted question from the QOLIBRI
Problems with sexuality	Adapted question from the QOLIBRI

HADS: Hospital Anxiety and Depression scale; PROs: patient-reported outcomes; PROMs: patient-reported outcome measures; PROMIS: Patient Reported Outcomes Measurement Information System; QOLIBRI: Quality of Life after Brain Injury questionnaire

In the original PROMIS short form for cognitive abilities, the phrase ‘as usual’ was often used in an item (e.g., ‘my memory has been as good as usual’). Due to the changes due to the aSAH itself, the reference point ‘as usual’ can be ambiguous; therefore, the working group decided to use a previously developed and translated customized Dutch form, which did not include ‘as usual’. Finally, we added 10 questions to the SOS-SAH to cover the nine remaining PROs. The working group rephrased an item of the QOLIBRI questionnaire, which was developed for patients with brain injury, for each of these PROs (the QOLIBRI item: ‘How bothered are you by problems with seeing or hearing?’).

#### Proxy measurements in the SOS-SAH

The working group considered proxy measurements relevant to assess cognitive abilities and changes in personality, as for these topics it was deemed most essential to gain an additional perspective. The PROMIS short form for cognitive abilities was adapted to fit a proxy perspective, with permission from PROMIS HealthMeasures.

#### Measurement protocol of the SOS-SAH

With regard to the timing of the use of the screening instrument, healthcare professionals suggested that the SOS-SAH screening should take place approximately six weeks after the aSAH treatment, since an early intervention in the case of persistent complaints might be of benefit to the patient. Some patients experienced deficits later in their recovery or did not request help at an early stage after their aSAH, however; therefore, both the healthcare professionals and patients recommended that the questionnaire should also be used later in the course of the aSAH recovery, such as six months after treatment.

#### Cognitive validation

Seven patients were interviewed to validate the SOH-SAH (Table 1). Additionally, we interviewed five family members to evaluate the proxy questionnaire.

Most of the questions from the existing PROMs were understood by the patients as intended, with only a few questions for which one or two patients had a different interpretation of the question. Most notably, patients found it hard to distinguish between ‘focusing’ and ‘concentrating’ (PROMS short form for cognitive abilities), while some patients answered the HADS question ‘I can enjoy a good book or radio or TV program’ (from its depression domain) negatively due to problems with their sight rather than feelings of depression. As a whole, however, the cognitive validation did not reveal any major problems for the content validity of the existing PROMs included in the SOS-SAH.

The 10 newly formulated items were also mostly understood as intended. Only one question about personality change was sometimes interpreted in a broader way, with patients taking changes of activities into account alongside personality change. However, these two may influence each other so this deviation was considered minor and the question was left unchanged.

#### Visual presentation

Healthcare professionals preferred a visual presentation of the SOS-SAH results that could be easily interpreted at a glance. They considered colored smileys for each PRO to be a good way to do this: a green smiley for a good outcome, orange for moderate, and red for a bad outcome. These categories were defined based on the cut-off values for the HADS [19] and the cut-off values for the PROMIS short forms based on T-scores [30]; the working group decided the cut-off values for the domains with one or two questions.

### Final result and recommended use

The SOS-SAH consists of 40 items covering 14 domains, with an additional section for a proxy measurement by family members containing nine items covering the cognitive abilities and personality change domains (Table 4). The result of the SOS-SAH can be summarized with a sum score, presented as a colored smiley for each domain.

**Table 4:** Definite version of the questions of the SOS-SAH

1	I have been able to bring to mind words that I wanted to use while talking to someone.	Not at all	A little bit	Somewhat	Quite a bit	Very much
2	I have been able to focus my attention.	Not at all	A little bit	Somewhat	Quite a bit	Very much
3	I have been able to remember to do things, like take medicine or buy something I needed.	Not at all	A little bit	Somewhat	Quite a bit	Very much
4	I have been able to think clearly.	Not at all	A little bit	Somewhat	Quite a bit	Very much
5	I have been able to remember the name of a familiar object.	Not at all	A little bit	Somewhat	Quite a bit	Very much
6	I have been able to concentrate.	Not at all	A little bit	Somewhat	Quite a bit	Very much
7	I have been able to pay attention and keep track of what I am doing without extra effort.	Not at all	A little bit	Somewhat	Quite a bit	Very much
8	I have been able to learn new things easily, like telephone numbers or instructions.	Not at all	A little bit	Somewhat	Quite a bit	Very much
9	In a busy environment I find myself quickly bothered by excessive stimuli.	Not at all	A little bit	Somewhat	Quite a bit	Very much
10	I feel tense or ‘wound up’.	Most of the time	A lot of the time	From time to time, occasionally	Not at all	
11	I still enjoy the things I used to enjoy.	Definitely as much	Not quite so much	Only a little	Hardly at all	
12	I get a sort of frightened feeling as if something awful is about to happen.	Very definitely and quite badly	Yes, but not too badly	A little, but it doesn’t worry me	Not at all	
13	I can laugh and see the funny side of things.	As much as I always could	Not quite so much now	Definitely not so much now	Not at all	
14	Worrying thoughts go through my mind.	A great deal of the time	A lot of the time	From time to time but not too often	Only occasionally	
15	I feel cheerful.	Not at all	Not often	Sometimes	Most of the time	
16	I can sit at ease and feel relaxed.	Definitely	Usually	Not often	Not at all	
17	I feel as if I am slowed down.	Nearly all the time	Very often	Sometimes	Not at all	
18	I get a sort of frightened feeling like ‘butterflies’ in the stomach.	Not at all	Occasionally	Quite often	Very often	
19	I have lost interest in my appearance.	Definitely	I don’t take so much care as I should	I may not take quite as much care	I take just as much care as ever	
20	I feel restless as I have to be on the move.	Very much indeed	Quite a lot	Not very much	Not at all	
21	I look forward with enjoyment to things.	As much as I ever did	Rather less than I used to	Definitely less than I used to	Hardly at all	
22	I get sudden feelings of panic.	Very often indeed	Quite often	Not very often	Not at all	
23	I can enjoy a good book or radio or TV programme.	Often	Sometimes	Not often	Very seldom	
24	I feel fatigued.	Not at all	A little bit	Somewhat	Quite a bit	Very much
25	I have trouble starting things because I am tired.	Not at all	A little bit	Somewhat	Quite a bit	Very much
26	How run-down did you feel on average?	Not at all	A little bit	Somewhat	Quite a bit	Very much
27	How fatigued were you on average?	Not at all	A little bit	Somewhat	Quite a bit	Very much
28	I have trouble doing all of my regular leisure activities with others	Never	Rarely	Sometimes	Usually	Always
29	I have trouble doing all of the family activities that I want to do	Never	Rarely	Sometimes	Usually	Always
30	I have trouble doing all of my usual work (include work at home)	Never	Rarely	Sometimes	Usually	Always
31	I have trouble doing all of the activities with friends that I want to do	Never	Rarely	Sometimes	Usually	Always
32	I am a different person than I was before the (subarachnoid) hemorrhage.	Not at all	A little bit	Somewhat	Quite a bit	Very much
33	How difficult do you find it to hold a conversation?	Not at all	A little bit	Somewhat	Quite a bit	Very much
34	How difficult do you find it to follow a conversation?	Not at all	A little bit	Somewhat	Quite a bit	Very much
35	How much difficulty do you have with your sight?	Not at all	A little bit	Somewhat	Quite a bit	Very much
36	How much difficulty do you have with your sense of taste?	Not at all	A little bit	Somewhat	Quite a bit	Very much
37	How much difficulty do you have with your sense of smell?	Not at all	A little bit	Somewhat	Quite a bit	Very much
38	How much difficulty do you have with your hearing?	Not at all	A little bit	Somewhat	Quite a bit	Very much
39	How bothered are you by headaches?	Not at all	A little bit	Somewhat	Quite a bit	Very much
40	Has the (subarachnoid) hemorrhage affected your sex life?	Not at all	A little bit	Somewhat	Quite a bit	Very much
**Proxy questions for family members**						
41	My family member is a different person than he/she was before the (subarachnoid) hemorrhage.	Not at all	A little bit	Somewhat	Quite a bit	Very much
42	My family member has been able to bring to mind words that he/she wanted to use while talking to someone.	Not at all	A little bit	Somewhat	Quite a bit	Very much
43	My family member has been able to focus his/her attention.	Not at all	A little bit	Somewhat	Quite a bit	Very much
44	My family member has been able to remember to do things, like take medicine or buy something he/she needed.	Not at all	A little bit	Somewhat	Quite a bit	Very much
45	My family member has been able to think clearly.	Not at all	A little bit	Somewhat	Quite a bit	Very much
46	My family member has been able to remember the name of a familiar object.	Not at all	A little bit	Somewhat	Quite a bit	Very much
47	My family member has been able to concentrate.	Not at all	A little bit	Somewhat	Quite a bit	Very much
48	My family member has been able to pay attention and keep track of what he/she is doing without extra effort.	Not at all	A little bit	Somewhat	Quite a bit	Very much
49	My family member has been able to learn new things easily, like telephone numbers or instructions.	Not at all	A little bit	Somewhat	Quite a bit	Very much

SOS-SAH: Questionnaire for Screening of Symptoms in aneurysmal Subarachnoid Hemorrhage

It was not possible for the majority of the interviewed patients to complete the PROM online without help, due to an inability to use a computer or a lack of energy to focus on a computer screen. The working group therefore recommends that, in practice, the PROM is presented to patients on paper.

The working group advises the following use of the SOS-SAH: the PROM could be employed multiple times during a patient’s follow-up care; for example, at six weeks, six months, and 12 months after the aSAH treatment. We advise healthcare professionals to discuss the results of the SOS-SAH with the patient to clarify whether the patient actually considers their condition to be a problem and to discuss whether there is a need for treatment. We suggest that patients are asked to prioritize the domains they want to discuss with the healthcare professional, in order to enhance patient engagement.

## Discussion

We describe the development of the SOS-SAH, a PROM for patients with successfully treated aSAH but with mild disabilities. The SOS-SAH screens for symptoms that often remain undetected because patients often do not actively mention these symptoms in consultations with healthcare professionals. The SOS-SAH was developed using domains and items from existing PROMs, with 10 newly formulated items. The use of existing PROMs offers the advantage of validated measurement properties, which increases the general acceptance of a PROM.

It is important to discuss the results of the PROM with patients. Pre-existing diseases or personal and environmental factors can influence impairments, limit activity, or restrict participation, as described in the ICF model [16]. These contextual personal and environmental factors are not captured in the SOS-SAH; therefore, we advise the healthcare professionals to ask patients for a clarification of their answers and discuss whether they would like to receive treatment.

The SOS-SAH covers cognitive complaints, but is not meant to replace formal cognitive tests. Subjective cognitive complaints are prevalent after subarachnoid hemorrhage [26]; however, cognitive complaints do not necessarily correspond with objective cognitive deficits. Previous research showed that cognitive complaints correlate not only with cognitive deficits, but also with depressive symptoms, anxiety, and coping style [26, 31–33]. Additionally, an aSAH can affect a patient’s ability to judge their own cognitive abilities [34]; therefore, patient-reported cognitive complaints may suggest cognitive dysfunctioning, but cannot replace formal neuropsychological tests. From a patient’s perspective, their own experience of cognitive impairments in daily life is what is most important and that is what must be treated. When the SOS-SAH reveals cognitive complaints, cognitive tests are advocated.

Both the anxiety and depression domains are incorporated in the SOS-SAH, although it might not be possible to measure these two as separate constructs. The HADS is a well-known and widely used health measurement instrument for measuring anxiety and depression; however, multiple studies have revealed it has an unclear factor structure [35]. The questions of the HADS are designed to avoid items that might arise due to physical rather than psychological states [36], but our cognitive validation interviews revealed that patients took physical symptoms into account while answering two questions of the HADS. We will evaluate whether this is problematic in future research on the SOS-SAH. Since our proposed use of the SOS-SAH is as a screening instrument in individual patient care, valuable information is gathered from the administration of the HADS.

The PROs that are incorporated in the SOS-SAH overlap with relevant PROs for patients after an ischemic stroke [37]; however, the most frequently occurring problems differ between patients treated for these two conditions. In patients after a stroke, the functional outcome is mainly driven by physical limitations due to focal brain damage, while for patients after an aSAH the main problems are usually cognitive disturbances, problems with societal participation, mood disturbances, and fatigue [37]. As a result of the higher age of patients after a stroke and the physical handicaps, the implications of these deficits in daily life might be less pronounced. It has not yet been investigated whether the clinical outcome of patients after an aSAH is different from the outcome of young stroke patients.

The results of a recently published systematic review show that the currently available PROMs are not sufficiently valid for use with patients successfully treated for aSAH [13], leading us to develop the SOS-SAH. The SOS-SAH differs from existing PROMs because the content validity has been evaluated thoroughly, and because it has been developed as a screening questionnaire and contains a proxy measurement. Furthermore, the SOS-SAH covers more topics than the SS-QoL, most notably anxiety, headache, smell, hearing, taste, and hypersensitivity to stimuli. In comparison with the SAHOT, the SOS-SAH is easier to use because its scores are not based on a Rasch analysis. Additionally, its face validity is better since the answer categories in the SAHOT do not contain the direction of change [13]. We therefore think that the SOS-SAH is more useful as a screening questionnaire in individual patient care.

### Strengths

The major strength of the development of the SOS-SAH is our extensive collaboration with patients and different types of healthcare professionals. Furthermore, the methodological adherence to the PROM-cycle and consensus-based standards for the selection of health status measurement instruments (COSMIN) is a strength [14, 38]. Lastly, validated PROMs available in multiple languages were included in the SOS-SAH, making the adaptation of the PROM for use in different countries highly feasible.

In qualitative research, data are gathered until data saturation is reached, and usually a large number of patients are interviewed to collect all important aspects of the outcome measure. In the development of the SOS-SAH, previously published PROs for patients after aSAH were used. These PROs were discussed in interviews with patients and healthcare professionals to decide on their relevance and whether they should be included in the SOS-SAH. This approach offers a scientific but pragmatic method to create a comprehensive overview of relevant PROs for patients. In doing so, we were able to interview a limited number of patients but still adhere to the COSMIN guidelines for PROM development [38].

### Limitations

The SOS-SAH does not cover all PROs that are important for patients with an aSAH, such as mobility and upper extremity function. This decision was made consciously in order to focus on undetected complaints and limit the length of the questionnaire. Additionally, we developed the SOS-SAH as a screening instrument for in-hospital care. The potential value of the SOS-SAH for use in other settings such as primary care or in the rehabilitation setting might be interesting for future research.

The optimal way of scoring PROMIS short forms is by the calculation of T-scores based on response pattern scoring instead of the T-scores in table form that we used. In response pattern scoring, the T-scores are calculated based on item parameters, i.e., item difficulty and discrimination of the item. It is only possible to accomplish this with an online scoring service in which all answers must be uploaded, which is less feasible in clinical practice.

Finally, the PROM has not yet been clinically tested, and thus is not yet validated. A validation study was recently begun.

## Conclusions

The development of a PROM for implementation in clinical practice for patients after an aSAH is described here. It resulted in the “Questionnaire for the Screening of Symptoms in aneurysmal Subarachnoid Hemorrhage” (SOS-SAH), which screens for self-reported symptoms of patients with mild disabilities. The SOS-SAH contains 14 domains and 40 items, in addition to a proxy measurement of nine items covering two domains.

We started a pilot study to test the feasibility of the SOS-SAH in daily clinical care to gather information about its practical use, to explore whether the information obtained from the SOS-SAH helps physicians and/or patients, and to determine its measurement properties.

## Data Availability

The authors are willing to provide non-identifying raw data related to this study upon request.
